# Three-dimensional water mapping of succulent *Agave victoriae-reginae* leaves by terahertz imaging

**DOI:** 10.1038/s41598-020-58277-z

**Published:** 2020-01-29

**Authors:** Abhishek K. Singh, Arely V. Pérez-López, June Simpson, Enrique Castro-Camus

**Affiliations:** 1 Centro de Investigaciones en Optica A.C., Loma del Bosque 115, Lomas del Campestre, Leon, Guanajuato 37150 Mexico; 2 Department of Plant Genetic Engineering, CINVESTAV Unidad Irapuato, Km. 9.6 Libramiento Norte Carretera Irapuato-Leon, Apdo. Postal 629, 36821 Irapuato, Guanajuato Mexico

**Keywords:** Biophysics, Plant sciences, Optics and photonics, Physics

## Abstract

While terahertz imaging has been used before for the determination of water content in vegetative tissue, most studies have either presented measurements of the temporal evolution of water content at a single-point of the plant or have presented two-dimensional images of leaves, demonstrating the potential of the technique, but relatively little of such information has been used to support biologically relevant conclusions. In this article we introduce terahertz time-domain spectroscopic imaging as a technique for the determination of the three-dimensional distribution of water in succulent plant tissues. We present the first three-dimensional water mapping of an agave leaf, which demonstrates an unprecedented capability to study the water retention mechanisms within succulent plants. We found that agave leaves are composed of a low-hydration outer tissue layer, defined by the outermost layer of vascular tissue that surrounds a high-hydration tissue, the carbohydrate rich hydrenchyma. The findings are supported by histological images and the correlation between the water content and carbohydrate presence is consistent with recently published findings of a remarkably large hydration shell associated with agave fructans.

## Introduction

Spectroscopic techniques, mainly in the infrared, visible and ultraviolet bands of the spectrum are common tools in contemporary plant science^1^. Yet, other bands of the electromagnetic spectrum remain relatively unexploited for the study of plants. While very well established technologies for producing and detecting microwaves and infrared waves have been widely available for well over a century, equivalent technology for the intermediate band, known as terahertz (THz) started its development in the late 1980s^2^. Only recently has plant science begun to incorporate this technique into its toolbox^3,4^. Since the chemical components of vegetative tissue have very interesting optical properties in the terahertz band, the technique could be adapted for a wide range of applications. For instance, sugars, proteins and other biomolecules that form tissues are relatively transparent to terahertz radiation, while, water is an extremely opaque substance at such wavelengths. Therefore, terahertz can be used as an excellent non-contact probe to determine water content in biological tissues^5,6^ and has been used in the past for the *in vivo* determination with unprecedented resolution of the water changes in *Arabidopsis thaliana*^7^. This application is particularly relevant given concerns on the effects of global warming on drought sensitive plant species that constitute the main food crops^8^. Another technique that has been used for the measurement of the spatial distribution of water in plants is Magnetic Resonance Imaging (MRI)^9^. Yet, MRI is based on the quantification of hydrogen spins in a sample, which are not only present in water, but in many biomolecules. In order to separate the contribution of water to the total magnetic resonance signal, a full multiple spin-echo experiment to determine the T_2_ decay has to be performed and multiexponential fitting of the signal needs to be done, which can be relatively inaccurate in many cases and limits the sensitivity of the technique. On the positive side, MRI is a non-destructive technique that can be applied *in-vivo*. That being said, non-destructive terahertz tomography is also possible in thin tissues as mentioned earlier and will be possible in the future for thicker tissues if the signal-to-noise ratio of the technique is increased.

Succulent plant species have adapted to survive under arid conditions by evolving the ability to store water within their tissues. At least 60 plant families are known to contain succulent species, including the Agavoideae sub-family of the Aspargaceae family, that includes agave species such as *A. tequilana*. Many aspects of the mechanisms of water retention of succulent plants remain a topic of intensive investigation. In particular the distribution of water within tissues and its correlation with micro and mesoscopic structures as well as the carbohydrate content of the tissue are subject to debate^10,11^. In order to test the capacity of terahertz radiation to contribute to the understanding of this subject, *Agave victoriae-reginae* plants were analyzed to determine their water content and localization. This species is characterized by rigid leaves with a prominent 3D anatomy, which accumulate carbohydrates in the form of fructan polymers. The leaves can easily be manipulated within the terahertz system making it an excellent model in which to implement the technique.

By employing the terahertz technique, 3D images of water distribution in *A. victoriae-reginae* leaves were generated and determination of water content (succulence). Terahertz was compared with traditional methods^10^ for calculation of this parameter. Additionally, a correlation between water distribution, leaf anatomy and carbohydrate localization in leaves of *A. victoriae-reginae* was determined by comparison of terahertz images with histological samples. This article, to the best of our knowledge, presents the first three-dimensional imaging of the water content in a plant leaf. The results represent a fundamental first step for the implementation of terahertz methodology in order to obtain a better understanding of the water distribution and retention mechanisms of succulent plants and how this correlates with drought resistance, anatomy, photosynthesis and other characteristics of the organism. The knowledge and the methodology could eventually be applied to the development of improved drought tolerance in crops such as maize or beans.

## Results

**Figure 1:**
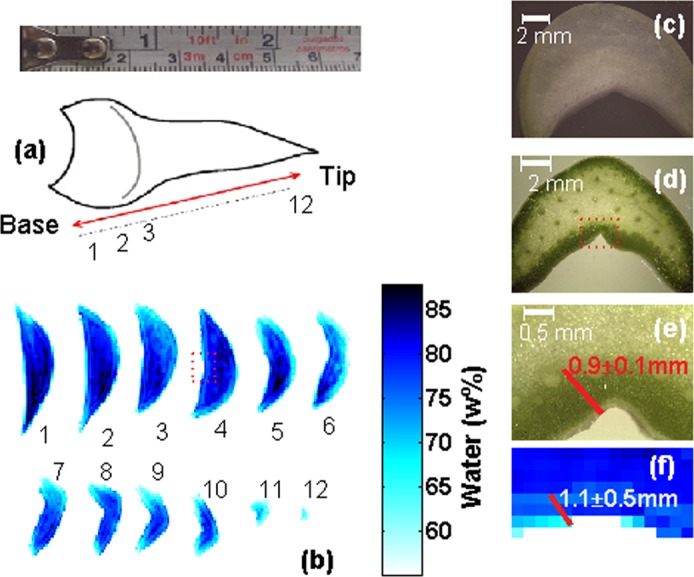
Determination of water content in leaf sections of *A. victoriae-reginae* leaves. (**a**) Approximate positions of sections 1–12 over the length of an *A. victoriae-reginae* leaf (**b**) Tomographic sections showing the water content distribution of an *A. victoriae-reginae* leaf. (**c**) Optical microscopy images of an *A. victoriae-reginae* basal (white) leaf section at 8X. (**d**,**e**) Bright light microscopy images of an *A. victoriae-reginae* middle (green) leaf section at 8X and 35X magnification respectively. The red box in (**d**), indicates the enlarged region shown in (**e**). (**f**) Enlarged terahertz image corresponding the box area of section 4 in (**b**).

As described in the methods, *Agave victoriae-reginae* leaves were removed from living specimens and immediately cut transversely to produce 12 sections in relation to the leaf axis as depicted in Fig. 1(a). From this initial dissection, we generated a collection of subsequent thin (~600 *μ*m) slices that were imaged using a terahertz time domain spectrometer. The data obtained from the collection of terahertz images were processed in order to generate a series of water content (*WC*) images, where the pixel-by-pixel amount of water was obtained. While all the water content images were obtained by terahertz transmission measurements as described in the methods section, the meaning of the water content presented from here on refers to the local weight fraction of water in the pixel and is denoted w%. This means that the images presented provide us with a measurement equivalent to cutting the tissue into very small volume “cubes”, then determining, for each cube, its fresh weight *W*_fresh_, dehydrating it, measuring its dry weight *W*_dry_ and obtaining the water weight percentage as

1 $$WC=\frac{{W}_{{\rm{fresh}}}-{W}_{{\rm{dry}}}}{{W}_{{\rm{fresh}}}}\times 100.$$

Individual tomographic water content images are shown in Fig. 1(b). These images allow us to clearly identify an outer, low water content layer, and an inner, more hydrated part of the leaf. It is also possible to observe that the water content shows additional degrees of variation over the cross section of the leaf and a gradual decrease along the leaf axis. The images show an outer layer of tissue with a hydration level between 60 w% and 70 w% (pale blue, Fig. 1b,f) that corresponds to the green, chloroplast containing chlorenchyma tissue shown in the tissue section in Fig. 1(d). In contrast, the internal region of the sections show a wider variation in water content above 70 w% (dark blue) with the highest level towards the center of the tissue sections. The regions of high water content correspond to the, visually, paler hydrenchyma tissue shown in Fig. 1(d,e). Furthermore as the area occupied by hydrenchyma decreases towards the leaf tip, water content also decreases. A good correlation in the thickness of the chlorenchyma was obtained when measurements were estimated from either the optical microscopy or the terahertz images (0.9 ± 0.1 mm and 1.1 ± 0.5 mm respectively) indicating that the terahertz images consistently reflect the histological micrographs. However, a strict relationship between the chlorenchyma and lower water content can not be inferred since the basal leaf section (Fig. 1b sections 1, 2) is devoid of active chloroplasts. Figure 1(c) shows the same water content pattern as green tissue (Fig. 1b sections 3–12). As observed, the low water content area reaches just beyond the outermost layer of vascular tissue and this may be the anatomical feature which determines the limit between low and high water content regions.

**Figure 2:**
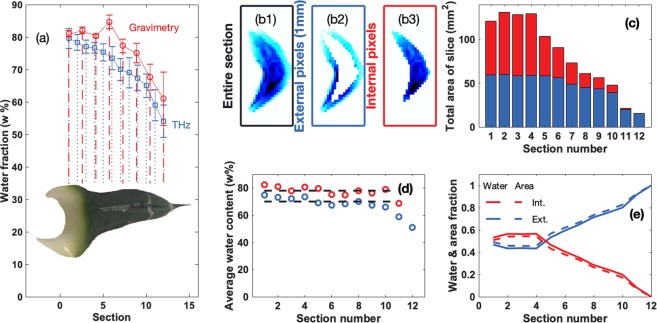
(**a**) Average water content for each leaf section of *A. victoriae-reginae*, comparing the determination of water by traditional methods (red) and by terahertz imaging (blue). (**b**1) Water mapping of a single section, the pixels in the external 1 mm layer (**b**2) and the pixels in the internal part (**b**3) respectively aiming to illustrate how each tomographic image was processed in order to obtain data presented in the following panels. (**c**) Area of each section along the leaf separating the contribution of the external, 1 mm layer (blue) and the internal pixels (red). (**d**) Average water content of the external (blue) and internal (red) pixels. (**e**) Fraction of the total water of a single section that corresponds to the external (continuous blue) and internal (continuous red) pixels, and fraction of the total area (dotted lines).

Succulence is traditionally calculated, as described in the methods section, by progressively dehydrating the plant tissue and determining the water loss. Figure 2(a) compares the results of the determination of water content in leaf sections using this traditional strategy with those obtained by terahertz imaging. By averaging the water content of each cross-section of the leaf we can obtain a measure of the succulence as a function of the position along the axis of the leaf. As shown in Fig. 2(a), the data obtained by traditional methods of succulence calculation (red line) correlate well with the succulence estimations obtained by terahertz imaging (blue line). The plot shows that the water content has a value close to 80 w% in the basal part of the leaf and decreases slowly for the first 9 to 10 slices after which water content decreases more rapidly towards the distal part of the leaf reaching about 55 w% near the tip. The measurements were performed on six different leaves from two different plants, three leaves from each. The values shown represent the average, while the error bars are the standard deviation for the six measurements.

Estimations of succulence at a higher resolution in different regions of a single leaf section would be extremely time consuming using traditional methods of calculation. However, performing image processing on the terahertz-based water images allowed us to carry out these estimations separately in the external leaf segments and the hydrenchyma. Figure 2(b1–b3) show how the images can be digitally dissected to separate the different types of tissue, allowing tissue specific estimates to be made. The pixels were classified in two types external, within 1 mm of the edge, shown in Fig. 2(b2) and internal, shown in Fig. 2(b3). Figure 2(e) shows the correlation between water content and area of each pixel type. As the area of leaf tissue occupied by hydrenchyma cells becomes smaller towards the leaf tip, the proportion of water in this tissue decreases in relation to that of the external tissue which, simultaneously, increases in area in relation to hydrenchyma towards the leaf tip. The total area of each section was quantified making a distinction of the relative area of the external (1 mm) layer and the internal, hydrenchyma region. This shows that over the first four sections the two kinds of pixels each account for approximately 50% of the area, as seen in Fig. 2(c). In the subsequent six sections the area contributed by the internal pixels decreases rapidly, while the contribution of the external layer only decreases marginally. The last two layers are composed only of external pixels, and the total area of the leaf decreases rapidly. In Fig. 2(d) we show the water content averaged separately over the external (as seen in Fig. 2b3) and internal pixels (as seen in Fig. 2b2) for each cross section, finding that the two types present a relatively constant, ~70 w% and ~78 w% water content respectively, with a relatively small fluctuation of ±3 w% around the average. Exceptions are the last two slices, where the external tissue suffers a rapid drop in water content, and hydrenchyma is no longer present. The relative contribution of each kind of pixel to the total water content for each slice is shown as a continuous line in Fig. 2(e). For the first four sections about 0.6 of the total water lies in the internal pixels, while for the following sections, the relative contribution of the internal pixels decreases, until all the water is stored in external pixels for the last two sections. This correlated very well with the relative fraction of area, depicted by dotted lines, and is consistent with the observation that the water content for each kind of tissue is nearly constant, at least for the first ten slices.

**Figure 3:**
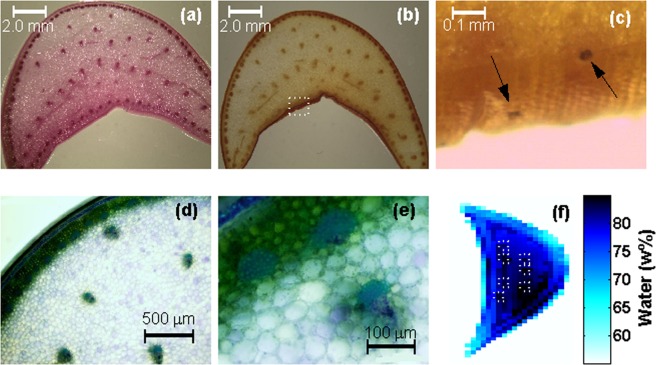
Carbohydrate staining of *A. victoriae-reginae* leaf cross sections. (**a**) Micrograph 8X of a leaf section after PAS staining. The pink coloring correlates with the presence of total carbohydrates. (**b**,**c**) Are 8X and 35X micrographs of lugol stained sections for starch identification. Panels (d,e) contain micrographs, highlighting the vasculature of the leaves. In (**f**) a closeup of section 2 from Fig. 1b, boxes were placed in order to identify areas of higher hydration that correlate with the position of the veins on the micrographs.

Agave species store carbohydrates in the form of fructans a trait that is thought to enhance their ability to withstand arid conditions by providing protection for cell membranes^12^. This hypothesis is supported by recent observations using terahertz spectroscopy that show a large hydration capacity for agave fructans^13^. In contrast starch is thought to accumulate only transiently in specific tissues^14^ and has not been associated with tolerance to dehydration. In order to explore the association between carbohydrates and water content in *A. victoriae-reginae* leaves, transverse sections of white basal leaf tissue were stained by either PAS (an non-specific stain for carbohydrates) or lugol (a specific stain for starch). As observed, PAS staining produces a pink coloration throughout the whole leaf section Fig. 3(a) indicating the presence of carbohydrates in general which could include both fructans and starch. However, with the exception of the stomatal guard cells, Lugol staining did not indicate the presence of starch in this tissue, as seen in Fig. 3(b,c). These results are consistent with previous observations obtained for *A. tequilana* and demonstrate the association of water retention with the presence of fructans in *A. victoriae-reginae* leaves^13^.

Toluidine blue staining of leaf sections Fig. 3(d,e) shows the distribution of vascular tissue within an *A. victoriae-reginae* leaf where closely associated vascular bundles are present around the periphery of the leaf whereas in the internal area they are more widely separated. The presence and distribution of vascular bundles can also be observed by the darker colored points indicated by white squares in Fig. 3(f).

**Figure 4:**
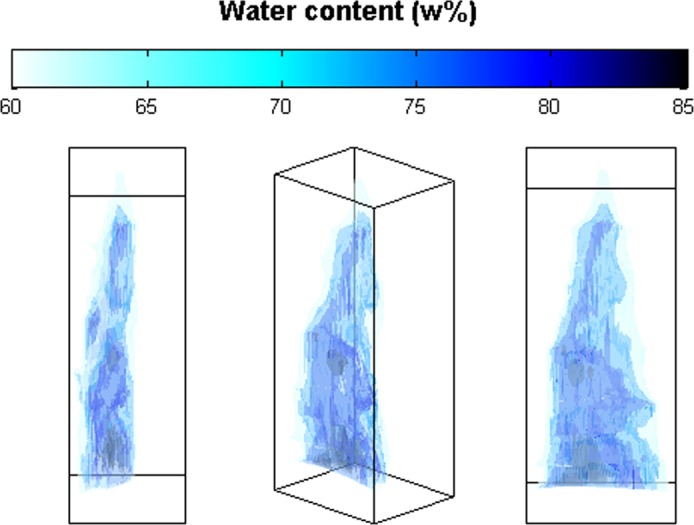
Three-dimensional representation of the water distribution in an *Agave victoriae-reginae* leaf (three perspectives presented). The image shows a relatively thin layer of low (<70 w%) water content tissue in the outside, pale blue, surrounding the more succulent part that appears in darker colors (>74 w%). The representation was generated by superimposing the 62 w%, 74 w%, 80 w% and 85 w% translucent isosurfaces. An animated version can be seen http://www.thz.org.mx/MuestrasIrapuato.php.

From the consecutive water content images it was possible to generate the 3-dimensional representation of water distribution in the leaf that is graphically represented in Fig. 4. The 3-dimensional representation shows a thin layer of low hydration (60–70 w%) tissue in the outer part of the leaf encompassing the more strongly hydrated tissue (>70 w%) that occupies the central section of the leaf. Darker lines/sections are also visible within the strongly hydrated central region indicating localized regions of even higher water content. Although the resolution is low these darker regions probably correspond to the vascular bundles shown in Fig. 3(d–f).

## Discussion

Terahertz tomography was used for the first time to determine the water content across sections of *Agave victoriae-reginae* leaves. Images clearly revealed different water content levels in the external and internal regions of the leaf. Although images and size measurements indicate that the external region corresponds closely to the photosynthetically active chlorenchyma, an identical pattern of water retention was observed in the, basal-leaves. This indicates that the presence of chloroplasts is not the determining feature in relation to water content. The relative amount of water contained in each of the two kinds of tissue for the different cross-sections is mostly due to the relative area occupied by each of them, and not by variations of their water retention capacity along the leaf, with the exception of the two distal slices, in which the chlorenchyma water content drops. However, this could also be caused by the surface-volume ratio in this region, which is relatively high in the vicinity of the leaf’s tip. This implies that the average distance to the stomata of any point inside the leaf is short, and therefore diffusion and water loss would be facilitated. It is also interesting to note that the external region also includes the outermost ring of vascular tissue and the density of vascular structures in this region is higher than in the central region of the leaf. This may indicate that in the external region, water transport, implying movement of water from surrounding cells and into vascular tissue, is more active in comparison to the central tissue. This leads to a lower water content in the outer region while water movement in the central region is lower, and therefore, higher levels of water are maintained. Based on the terahertz sections the water content across the length of the leaf could be determined. The measurements show it to be relatively constant until reaching the sections closest to the leaf tip where water content dropps, consistent with a reduction in the size of the internal region.

The presence of fructans in succulent leaf tissue agrees with the hypothesis that fructans play a role in tolerance to low water conditions^12,15–18^, since they have the ability to stabilize cellular membranes^12^. Furthermore, a recent study using terahertz spectroscopy that demonstrated that agave fructans have an outstanding hydration capacity^13^. It was shown that agave fructans, with a degree of polymerization of ~22, form hydration spheres containing of the order of 300 water molecules, which is remarkably high in comparison to most sugars.

When water content of the leaf section determined by terahertz imaging was compared to the traditional methods used to calculate water content or succulence a very good correlation was obtained, while the measurements determined for external and internal regions were also very consistent between the terahertz and the histological images indicating that terahertz imaging provides a faithful representation of the leaf tissue sections. Furthermore terahertz imaging presents additional advantages for water content determination in comparison to traditional methods. For example the leaf sections are simply cut and subjected to imaging whereas in the traditional method, sections must be weighed, dried for up to several days and reweighed before water content can be determined. In addition, “digital dissection” by image processing of different regions of each individual section can be carried out with ease based on the terahertz images. Whereas this would be extremely time consuming and imprecise, if not impossible, using traditional methods. Finally for the first time a three dimensional water-content image of a complete *Agave victoriae-reginae* leaf was generated by combining the terahertz images, clearly indicating the high water content “central core” surrounded by the external region with lower water content. Although it is unclear under this resolution, the darker linear regions observed in the internal core could correspond to vascular tissue.

## Conclusions

In conclusion we have presented a terahertz-imaging based study of the three-dimensional distribution of water in agave leaves which allowed us to rapidly and accurately determine water content levels in specific areas of the *Agave victoriae-reginae* leaf. The elevated presence of fructans in the succulent tissue suggests that those carbohydrates might be, at least partly, responsible for its relatively high water retention capacity. This is, to the best of our knowledge, the most detailed study ever presented of the water content distribution in succulent plants. The technique presented here has many advantages in comparison to the methods previously used to determine succulence. The results open new and exciting possibilities for future studies of succulence in plant tissues and also for the development of high throughput phenotyping systems that would represent an enormous advantage for breeding programs focused on drought tolerant crops. For further basic studies, comparison of water content, localization and transport in different agave varieties would be of much interest. In addition, improved resolution of the terahertz images will reveal additional information of relevance.

## Methods

### Plant material

The biological materials used were *Agave victoriae-reginae* plants of around 4 years of age, which had been grown in a greenhouse at CINVESTAV Unidad Irapuato, plants were originally obtained from the Botanical Garden”El Charco del Ingenio”, San Miguel de Allende, Guanajuato México in 2017. These were maintained in pots of 3500 *cm*^3^, with a soil mixture of fine tezontle and leaf litter $\mathrm{(50:50}v/v)$ and were watered once a week with ~500 *cm*^3^.

### Leaf processing for micrographs

Leaf samples were manually excised from the plants, following the rosette formation. Fresh tissue was washed and then cooled to 4° to be sectioned with a razor blade by hand in sections of ~50 *μm*, to be immediately stained.

Toluidine blue staining was used to differentiate tissue structures based on^19^. Following the sectioning, samples were drawn in 1 mL of toluidine blue solution (0.05% w/v in phosphate buffer 1 M at pH 6.8) for 3 min, then washed for 1 min in deionized water and visualized under bright field microscopy (OLYMPUS BX60 microscope and Leica E24HD stereoscope). To identify total carbohydrates on fresh tissue sections, Periodic Acid-Schiff (PAS) and Lugol staining were carried out and visualized as described in^14^.

Measurements of agave leaves such as weight, volume and density of excised tissues were registered. Excised leaves were dried at 60° for 10 days (Boekel oven), and the loss of water was monitored every 24 h, until its constant weight. A water displacement method to determine the volume of agave leaves was used in a graduated cylinder before and after the drying process. Water content was experimentally determined (Eq. 1) while density [*g*/*cm*^3^] was calculated as follows:

2 $${\rho }_{{\rm{leaf}}}=\frac{{W}_{{\rm{dry}}}}{{V}_{{\rm{dry}}}}$$

### Terahertz time-domain spectroscopy

**Figure 5:**
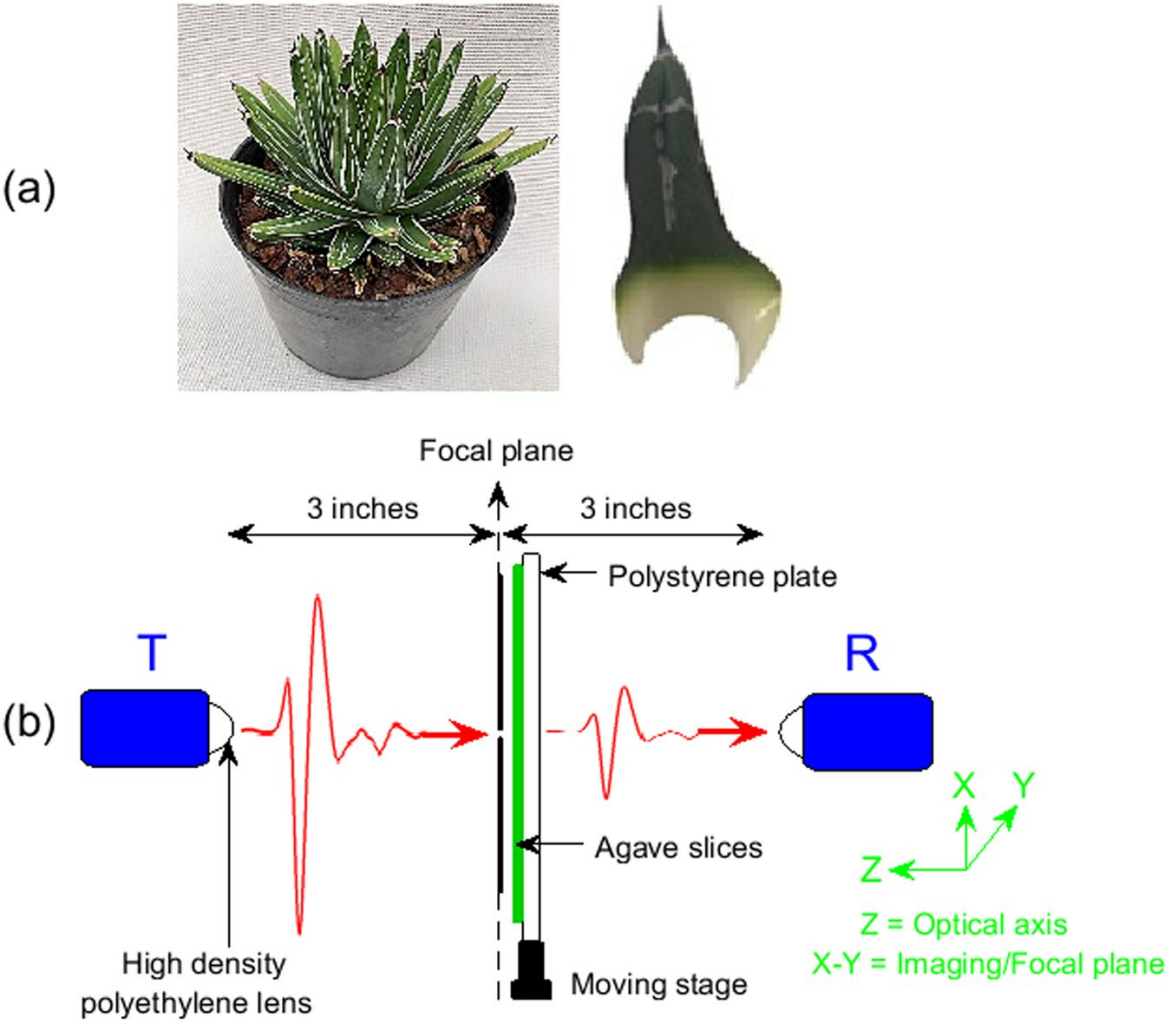
(**a**) Images of *Agave victoriae-reginae* plants, and a typical leaf similar to the ones used in this study; (**b**) Terahertz time-domain imaging set-up in transmission mode. The transmitter, pinhole and receiver fall on the same optical axis. The sample slices were mounted on a moving stage that raster-scanned the samples across the XY plane next to pinhole.

Measurements were performed on leaves from *Agave victoriae-reginae* plants as shown in Fig. 5(a). Given the fact that water is a highly absorptive liquid at terahertz frequencies, the transmitted intensity of the terahertz radiation is a function of the water content of the plant tissue. Since the agave leaves are significantly thicker than the leaves from typical plants, we performed measurements on thin 0.6 mm transverse sections of the agave leaves. Measurements were performed on 6 leaves in total, with 3 leaves each from two different plants.

The time-domain terahertz imaging measurements were performed with a API TeraGauge spectrometer, consisting of a femtosecond fiber laser coupled to photoconductive switches that acted as emitter and detector of terahertz pulses spanning over the frequency range of 0.1–2 THz. The measurements were performed with the terahertz imaging systems configured in transmission geometry, as shown in the Fig. 5(b). A 1.5 mm pinhole was mounted at the focal plane of the transmitter in order to improve the image resolution. The terahertz transmitter, pinhole, and receiver were aligned on a common optical axis. All the measurements were performed with pixel size and scanning speed of 0.5 mm and 0.5 mm/sec, respectively. The agave slices were mounted next to the pinhole, on a moving XY stage held on a polystyrene foam plate, as shown in Fig. 5.

### Terahertz signal and image processing

**Figure 6:**
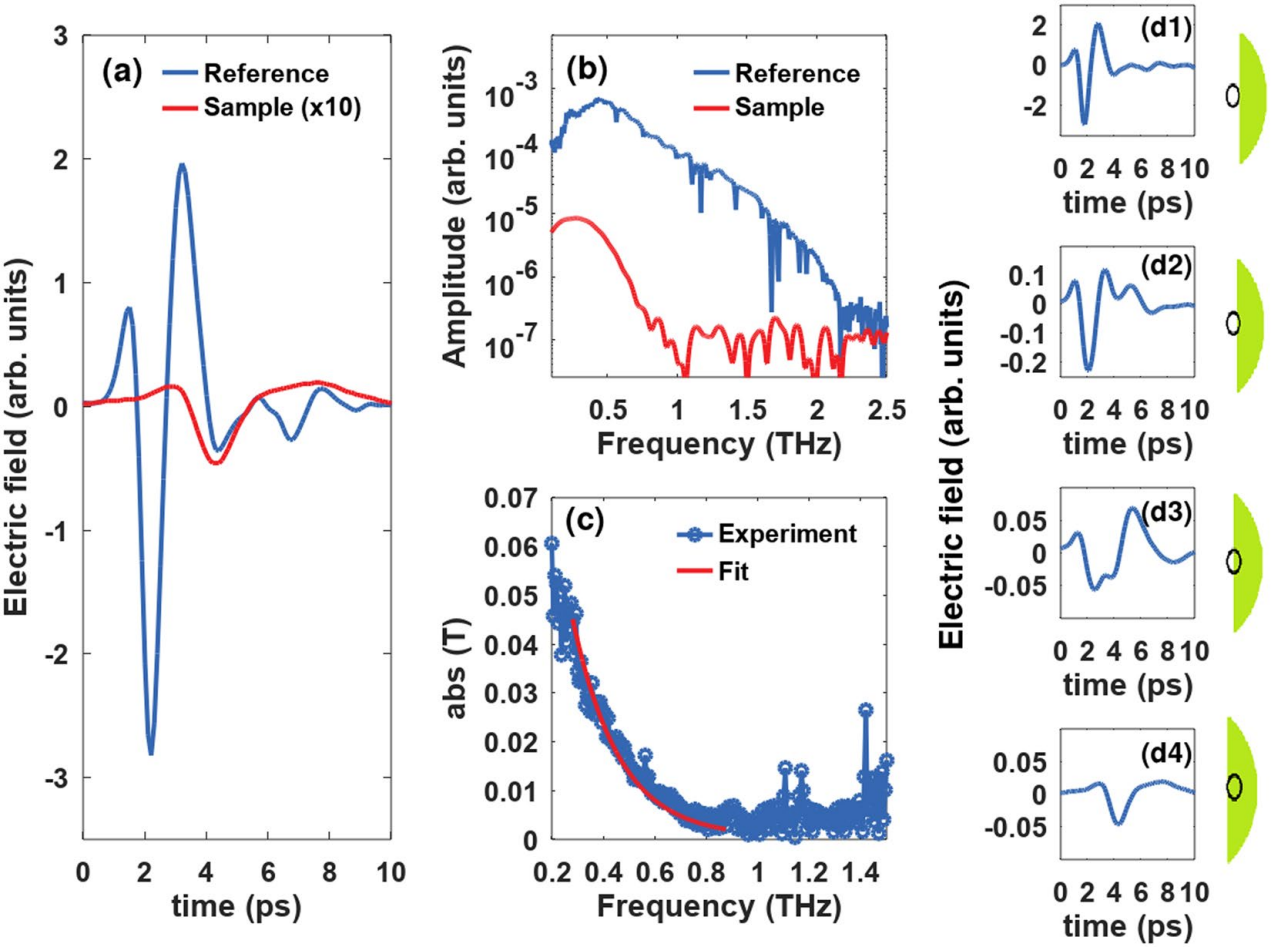
(**a**) Terahertz time-domain reference signal and the signal transmitted through hydrated agave slice; (**b**) Frequency domain electric field amplitude of reference signal and signal transmitted through hydrated slice; (**c**) Experimental values of the transmission amplitude (blue dots) and fit using the model (red line). (**d**1–4) Terahertz waveforms measured passing through the pinhole while scanning across the edge of the leaf. The signals (**d**1,**d**4) corresponds positions where pinhole is either completely outside the sample or completely inside the sample, respectively. The signals (**d**2,**d**3) are positions where the signal was partially transmitted through the sample and partly through air. The schematics shown on the right of each subfigure depict the position of the pinhole for each measurement. In order to prevent inaccurate measurements all pixels that did not comply with full transmission through the sample were discarded.

In order to process the terahertz images and estimate the pixel-by-pixel water content, we used an effective medium theory model to fit the transmission spectra of each pixel. In this section we will only give a brief description of the signal processing, yet, for further details on the processing please consult ref. ^6^. According to the Landau-Lifhitz-Looyenga model, the complex dielectric function of a mixture can be expressed as

3 $$\sqrt[3]{{\varepsilon }_{L}(\omega )}={x}_{W}\sqrt[3]{{\varepsilon }_{W}(\omega )}+{x}_{S}\sqrt[3]{{\varepsilon }_{S}(\omega )}+{x}_{A}\sqrt[3]{{\varepsilon }_{A}(\omega )},$$

where *ε*_*k*_ are the dielectric functions of the mixture components and *x*_*k*_ are the relative volumetric fractions. The indices refer to leaf (L), water (W), solid/dry tissue (S) and air (A), respectively, and *ω* is the frequency. The transmittance is given by ratio of the Fourier transform of the pulse transmitted through the sample divided by the Fourier transform of the reference, in the absence of the sample, ${T}^{i,j}(\omega )={E}_{sam}^{i,j}(\omega )/{E}_{ref}^{i,j}(\omega )$, where the indices *i* and *j* identify the pixels of the image. Since the transmittance can be also written as function of the complex refractive index $\tilde{n}=\sqrt{{\varepsilon }_{L}}$ of the leaf

4 $${T}^{i,j}(\omega )={t}_{12}^{i,j}(\omega ){t}_{21}^{i,j}(\omega ){e}^{i\frac{\omega d}{c}({\tilde{n}}^{i,j}(\omega )-1)},$$

which are at their time, according to the equation a function of the water content, where ${t}_{12}^{i,j}(\omega )$ and ${t}_{21}^{i,j}(\omega )$ are the Fresnel coefficients of the air-leaf and leaf-air interfaces, and *d* is the thickness of the sample. In order to calculate the water fraction for each pixel from the terahertz signal, we use the known input parameters ${\varepsilon }_{W}(f)$, ${\varepsilon }_{S}(f)$, ${\varepsilon }_{A}(f)$, and perform iterations over the parameters *x*_*W*_, *x*_*S*_, *x*_*A*_, in order to get the best possible fit to the transmittance. An example of the electric field for the reference signal and the signal transmitted through a hydrated sample, in time and frequency domains, are shown in Fig. 6(a,b), respectively. A quantitative fit of the transfer function to the model is shown in Fig. 6(c) for the frequency region of 0.3–0.9 THz, which was chosen because that band shows the highest signal-to-noise ratio in our measurements.

### Edge pixel removal

As shown in Fig. 5(b), a 1.5 mm pinhole was used in order to improve image resolution. The terahertz images were performed with pixel size 0.5 mm determined by the raster scanning stages. While scanning through the edges, there are 3–4 pixels in which the terahertz signal transmits partially through the sample and partly through air, as seen in Fig. 6d1–d3. In order to make sure that only those pixels where the signal was transmitted completely through the sample were considered, such as in Fig. 6d4, we discarded pixels where the signal was inconsistent with transmission only through the sample.
